# Effects of early afterdepolarizations on excitation patterns in an accurate model of the human ventricles

**DOI:** 10.1371/journal.pone.0188867

**Published:** 2017-12-07

**Authors:** Enid Van Nieuwenhuyse, Gunnar Seemann, Alexander V. Panfilov, Nele Vandersickel

**Affiliations:** 1 Department of Physics and Astronomy, Ghent University, Ghent, Belgium; 2 Institute for Experimental Cardiovascular Medicine, University Heart Center Freiburg, Bad Krozingen, Germany; 3 Faculty of Medicine, University of Freiburg, Freiburg, Germany; Georgia State University, UNITED STATES

## Abstract

Early Afterdepolarizations, EADs, are defined as the reversal of the action potential before completion of the repolarization phase, which can result in ectopic beats. However, the series of mechanisms of EADs leading to these ectopic beats and related cardiac arrhythmias are not well understood. Therefore, we aimed to investigate the influence of this single cell behavior on the whole heart level. For this study we used a modified version of the Ten Tusscher-Panfilov model of human ventricular cells (TP06) which we implemented in a 3D ventricle model including realistic fiber orientations. To increase the likelihood of EAD formation at the single cell level, we reduced the repolarization reserve (RR) by reducing the rapid delayed rectifier Potassium current and raising the L-type Calcium current. Varying these parameters defined a 2D parametric space where different excitation patterns could be classified. Depending on the initial conditions, by either exciting the ventricles with a spiral formation or burst pacing protocol, we found multiple different spatio-temporal excitation patterns. The spiral formation protocol resulted in the categorization of a stable spiral (S), a meandering spiral (MS), a spiral break-up regime (SB), spiral fibrillation type B (B), spiral fibrillation type A (A) and an oscillatory excitation type (O). The last three patterns are a 3D generalization of previously found patterns in 2D. First, the spiral fibrillation type B showed waves determined by a chaotic bi-excitable regime, i.e. mediated by both Sodium and Calcium waves at the same time and in same tissue settings. In the parameter region governed by the B pattern, single cells were able to repolarize completely and different (spiral) waves chaotically burst into each other without finishing a 360 degree rotation. Second, spiral fibrillation type A patterns consisted of multiple small rotating spirals. Single cells failed to repolarize to the resting membrane potential hence prohibiting the Sodium channel gates to recover. Accordingly, we found that Calcium waves mediated these patterns. Third, a further reduction of the RR resulted in a more exotic parameter regime whereby the individual cells behaved independently as oscillators. The patterns arose due to a phase-shift of different oscillators as disconnection of the cells resulted in continuation of the patterns. For all patterns, we computed realistic 9 lead ECGs by including a torso model. The B and A type pattern exposed the behavior of Ventricular Tachycardia (VT). We conclude that EADs at the single cell level can result in different types of cardiac fibrillation at the tissue and 3D ventricle level.

## Introduction

Cardiac contraction is initiated by electrical waves of excitation. Interruption of the normal spreading of the excitation waves, can result in cardiac arrhythmias or sudden cardiac death (SCD) [1]. As SCD is one of the main causes of death in the industrialized world, understanding the mechanisms of cardiac arrhythmias is of great interest in cardiology. One of these mechanisms is linked to the abnormal time course of the action potential (AP) of the cardiac cells called early afterdepolarizations (EAD). An EAD is defined as a reversal of AP before the completion of its repolarization phase [2–4] and emerges due to an imbalance of outwards and/or inwards oriented currents in the plateau phase of the AP. This imbalance can be the result of genetic defects such as the long QT syndrome [5–7], they can appear due to the action of pharmacological agents [8, 9], or they can be present in several other conditions [10, 11]. So far, EADs have been widely studied at the single cell level both experimentally and theoretically [10, 12]. However, the most important question is how EADs result in cardiac arrhythmias and it is important to study them at the tissue and 3D ventricles level. Studies in 2D have shown that EADs can affect spiral wave dynamics [13–15] as they can cause meandering of the spiral wave. It was also shown that EADs can cause purely focal activity [16–20]. In [21, 22] 2D complex behavior due to EADs was studied in two different human ventricular models, namely the Ten Tusscher Panfilov (TP06 [23, 24]) and the Ohara-Rudy model (ORD [25] model) for a wide range of parameter values. Also in 2D heterogeneous tissue, EADs were investigated [26–30].

However, EAD dynamics at the 3D ventricles was not yet properly addressed. In [16], spatial EAD activity was studied in an anatomical model of a homogeneous rabbit heart. It was found that EADs lead to ectopic activity whereby EADs locally cluster. These local clusters randomly move in time and space thereby creating an ECG similar to that of polymorphic ventricular tachycardia (PVT). EADs in anatomical models of human heart were presented only in two papers. First, in [31] islands of M-cells were added based on sizes of Glukov et al. [32]. The authors modeled conditions of the LQT3 syndrome by enhancing the late Sodium current and obtained ECG similar to PVT of the Torsade de Pointes (TdP) type produced by a moving spiral wave. In [30] the effect of EAD-prone heterogeneities was studied. It was found that reducing the RR in these heterogeneities in comparison with the surrounding tissue can lead to ectopic activity in these heterogeneities, whereby these sources can be competing, leading to TdP like ECGs. However, so far, there were no studies aiming to quantify and classify possible excitation patterns which can occur at the 3D ventricles level in wide range of parameters, as was done in 2D in [21, 22]. The patterns at the 3D ventricles level may differ substantially from those in 2D as wave propagation in the 3D ventricles is a 3D process which occurs in anisotropic tissue and has certain spatial limitations.

The aim of this paper was therefore to perform a detailed study of excitation patterns in the TP06 model in an anatomical model of the human heart without electrophysiological heterogeneities. Similarly as in [21], we have induced EADs by gradually reducing *I*_*Kr*_ and increasing *I*_*CaL*_, and performing a full 2D-parametric study. We also simultaneously computed realistic 9-lead ECGs in a torso model [33], which was used for the interpretation of the results.

## Materials and methods

### Mathematical model for human endocardial ventricular tissue in 3D

For this study, we used a modified version of the TP06 model for human ventricular endocardial cells [23, 24]. The single cell membrane potential is described by the ordinary differential equation:

$$\frac{dV_{m}}{dt}=-I_{ion}-I_{stim}$$ (1)

where *V*_*m*_ is the voltage, *t* is time, *I*_*ion*_ the sum of all trans membrane ionic currents in units of $\frac{pA}{pF}$, *I*_*stim*_ the externally applied stimulus current in units of $\frac{pA}{pF}$ and *C*_*m*_ the capacitance per unit of surface area. *I*_*ion*_ is given by:

$$I_{ion}=I_{Na}+I_{K1}+I_{to}+I_{Kr}+I_{Ks}+I_{CaL}+I_{NaCa}+I_{NaK}+I_{pCa}+I_{pK}+I_{bCa}+I_{bNa}$$ (2)

where *I*_*Na*_ is the Sodium current, *I*_*K*1_ the inward rectifier Potassium current, *I*_*to*_ the transient Potassium outward current, *I*_*Kr*_ the rapid delayed rectifier Potassium current, *I*_*Ks*_ the slowly delayed rectifier Potassium current, *I*_*CaL*_ the L-type Calcium current, *I*_*NaCa*_ the Sodium-Calcium exchanger current, *I*_*NaK*_ the Sodium-Potassium exchanger current, *I*_*pCa*_ and *I*_*pK*_ Calcium and Potassium plateau currents and *I*_*bCa*_ and *I*_*bNa*_ Calcium and Sodium background currents. The behavior of these channels is based on a wide range of human-based electrophysiological data. Specific details can be found in [23, 24].

The two currents which we varied in this study were *I*_*CaL*_ and *I*_*Kr*_. In the modified version of the TP06 model, we implemented a twofold decrease of the *f*-gate time constant of the implementation of *I*_*CaL*_. This resulted in an increased probability of EAD formation [21, 34]. In order to compensate for this change, we increased the default TP06 value of the maximal conductance *G*_*CaL*_ by 2, resulting in a single cell AP comparable to the original model. Hereafter, our adapted model is called the default one.

The spatial-temporal evolution of the membrane potential *V*_*m*_ in a 3D tissue was governed by the partial-differential equation (PDE)

$$\frac{\partial V_{m}}{\partial t}=-I_{ion}+\sum_{i,j=1}^{3}\frac{\partial }{\partial x_{i}}D_{ij}\frac{\partial V_{m}}{\partial x_{j}}$$ (3)

The conductivity tensor *D*_*ij*_ was calculated from the fiber orientation field using the formula

$$D_{ij}=D_{l}\delta_{ij}+\left(D_{l}-D_{t}\right)\alpha_{i}\alpha_{j}$$ (4)

where $D_{l}=0.00154\frac{cm^{2}}{ms}$ accounts for the conduction in the longitudinal direction. We used a 2: 1—ratio for the conduction velocity anisotropy, based on experiments ([35]) and hence a ratio of 4: 1 for *D*_*l*_: *D*_*t*_. With these values we obtain a maximum planar CV of around 70 cm/s in the fiber direction, in agreement with experimentally reported values of the conduction velocity in human ventricular tissue [35]. Based on these parameters and the integration of Eq 3, we find a spatial constant around 555*μm* which is consistent with experimental values [36]. Also, using this model, we simulated a pattern of normal propagation. We excited the tissue in locations corresponding to regions of exit of the wave from the Purkinje system to the ventricles [37]. The results are shown in S1 Fig. We can see that total excitation of the 3D ventricles required 0.096 s which corresponds to experimentally found duration of the QRS complex. Our results correspond to physiological changes by reducing/blocking the *I*_*Kr*_ current by e.g. Dofetilide, a *I*_*Kr*_ blocker. Large changes in increase of *I*_*CaL*_ can be achieved by applying Isoproterenol, which can enhance the L-type Ca current in the range from 1.5- to 8-fold, see [34].

### Numerical methods

To integrate the PDE (Eq 3), all calculations were run on a NVIDIA-CUDA GPU. We used a finite difference numerical integration method with a time step of 0.02 ms and a space step of 400 *μm*. In most of our simulations we used a spatial discretization step of 0.4 mm. Although such spatial resolution was previously used in several studies involving anatomically accurate modeling [38, 39] it is rather coarse for most 2D studies involving ionic models of cardiac tissue [40]. Therefore, in order to test the effect of the spatial discretization, we performed additional simulations for the A, B and O patterns with a space step of 200 *μm* and compared the results with a space step of 400 *μm* in an anisotropic wedge preparation. The wedge represented a rectangular slab of cardiac tissue with dimensions 8 x 4 x 4 cm. We also included realistic fiber orientations from -60 degrees at the epicardium to 60 degrees at the endocardium. The results of these simulations for representative values of the parameters are shown in the S2 Fig. We see that the qualitative pattern of excitation for the spatial resolution of 0.2 mm and 0.4 mm are similar. In addition, also the temporal Fourier transform which was used as the main determinant to distinguish different regimes, was similar for 0.2 mm and 0.4 mm spatial resolution for both A and B patterns. Finally, we also tested the existence of phase waves, and we found the same result for 0.2 mm and 0.4 mm spatial resolution for all A, B and O patterns. Therefore, we conclude that our main results are not likely to be affected by using larger spatial discretization step. All codes were programmed in c, c++ and Python. Similar to [21], 2 protocols to induce the excitation patterns were used: burst pacing (multiple S1 pacings) and an S1S2 protocol for the creation of a spiral (scroll) wave in the 3D ventricles [41]. First, for the burst pacing protocol, each time a certain point was below -60 mV a new S1 pulse was applied, this during the first 4 s of the simulation. Hence, the pacing frequency was determined by the AP duration (APD). The amount of paces in these 4 s varied depending on the parameter values. The probed parameter range determined by a 6.0- to 14.0-fold of the default value of *G*_*CaL*_ corresponding with a physiological range of 3–7 times normal *G*_*CaL*_ and a 0.0- to 1.0-fold of the default value of *G*_*Kr*_. Afterwards, another 2 s of each parameter value was simulated to let the patterns evolve without further pacing. Second, for the same parameter range we initiated a scroll wave with the S1S2 protocol. Sometimes the protocol failed and we created a spiral by slowly changing the parameters from neighboring parameters which were successful for the S1S2 protocol. In this way, we were able to cover the desired parameter range. Each S1S2 pattern was simulated for 5s.

### Filament detection with phase mapping

In conditions with a normal AP, the location of the filament or a scroll wave could be defined by intersection of a certain isopotential line and the zero derivative line [42, 43]. However, in this study, APs were often disturbed by EADs, making the AP derivative and the isovoltage line ambiguous. To find filaments for EAD disturbed tissue, we used the techniques presented in [44–47]. The basic idea was to find the phase singularities in the tissue. In order to create the phase space in which we could find the topological defects, or singularities, we created a second time series namely the Hilbert transform of the AP. At first, we smoothed our data by using a 3rd order polynomial and a 102 ms time frame. Then, we calculated the relative minima and maxima of the smoothed AP and connected those points with a piecewise cubic Hermite polynomial. The average of those two data sets was then subtracted from the AP in order to get an AP course around a more localized equilibrium. From this modified AP, we calculated the Hilbert transform. Due to these modifications on the AP, angles could be uniquely defined in the EAD-regions, as the equilibrium of both the Hilbert transform and the modified AP now plays the role of attractor in phase space determined by AP and Hilbert transform. The time-course of this angle was equal to the Hilbert transform and values ranged from -*π* to *π*. To determine the singularities with the convolution kernels (Sobel operator) presented in [44–46], we sliced the 3D ventricles in 3 orthogonal directions every other 5 node points of the 3D ventricles. Each of these sliced results was combined to cover the 3D ventricles and every point differing from zero was considered as a phase singularity. The location of the filaments could change over time, and hence the points were followed. As additional constraint, we defined it as a true filament only if a collective of points lived longer than one circulation (200ms). The singularities were detected in the last second of the simulation but were only followed in the interval [200ms, 800 ms] of this second to avoid any edge-effects of the time series manipulations.

### ECG calculations

ECGs were calculated using the lead field method. For this, a lower resolution tetrahedral mesh of the 3D ventricles embedded in the torso was used. Independently, all elements of the tetrahedral 3D ventricles mesh were set to a value of 1 and a field calculation based on the bi domain theory was performed on the torso (details can be found in [33, 48]). The result for each electrode position was stored in a vector. All resulting solution vectors are than combined into the N x M lead field matrix consisting of N 3D ventricles elements and M lead positions. For the simulations, the source component based on the gradient of the trans membrane voltage just have to be interpolated onto the lower resolution tetrahedral mesh and multiplied by the lead field matrix to generate the ECG on the given lead positions.

## Results

**Fig 1:**
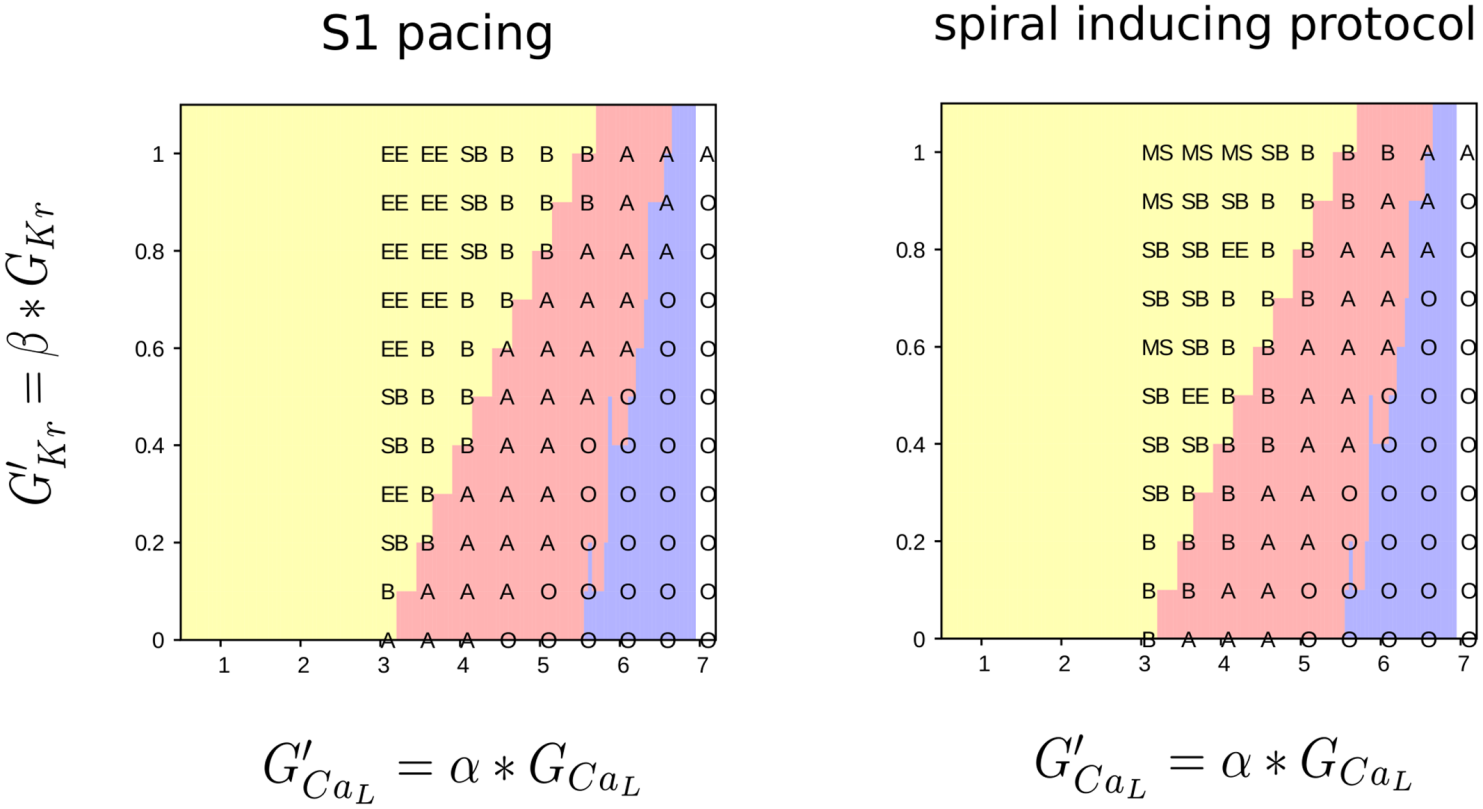
Results upon homogeneously reducing repolarization reserve in the 3D model. We reduced *I*_*Kr*_ and increased *I*_*CaL*_ for the two different protocols: burst pacing (left) and spiral wave induction (right) and observed the different resulting excitation patterns. We found 6 different possible types: spiral fibrillation type B (B), spiral fibrillation type A (A), Oscillatory type (O), spiral break-up (SB), meandering spiral (MS) and non-sustained patterns (EE, excitation ended). Background colors refer to the single cell results. Yellow represents normal action potential. Red denotes action potentials with EADs but in which the cells still recover to resting state potential. Blue are regions in which single cells fail to repolarize and oscillate around a high voltage equilibrium.

To generate the excitation patterns, we used two stimulation protocols: 1) the burst pacing protocol in which complex patterns are self induced due to tissue properties and 2) the S1S2 stimulation protocol where an initial spiral wave was setup by a proper choice of initial conditions. For both initial conditions, RR was gradually changed by variation of *G*_*CaL*_ and *G*_*Kr*_. In this parameter regime, we observed several types of excitation patterns which are characterized in detail in the next section. Here we just list them. For the burst pacing protocol, we found 4 different excitation patterns: spiral fibrillation type B (B), spiral fibrillation type A (A), an oscillatory excitation (O) and a spiral breakup region (SB), see Fig 1. If no EADs emerged, resulting in no pattern formation, excitation ended (EE) after the last applied stimulus. In addition, for the S1S2 protocol, we also found stable spirals (S) and meandering spirals (MS). In this paper however, we focused on the B, A and O excitation patterns. S, MS and SB patterns will be addressed in future study. The established patterns of excitation did not depend on the initial conditions. In case after the 5 (s1s2) and 6 (burst pacing) seconds of simulation the patterns still differed, we created longer simulations of 10 seconds. We observed that at end of our 10 second simulations the excitation patterns created by s1 pacing and s1-s2 stimulation for the same parameter values were similar in visual examination and produced the same temporal Fourier transform for B and A excitation patterns. Similar result was also shown previously in [21] for 2D simulations.

### Type of the excitation patterns

**Fig 2:**
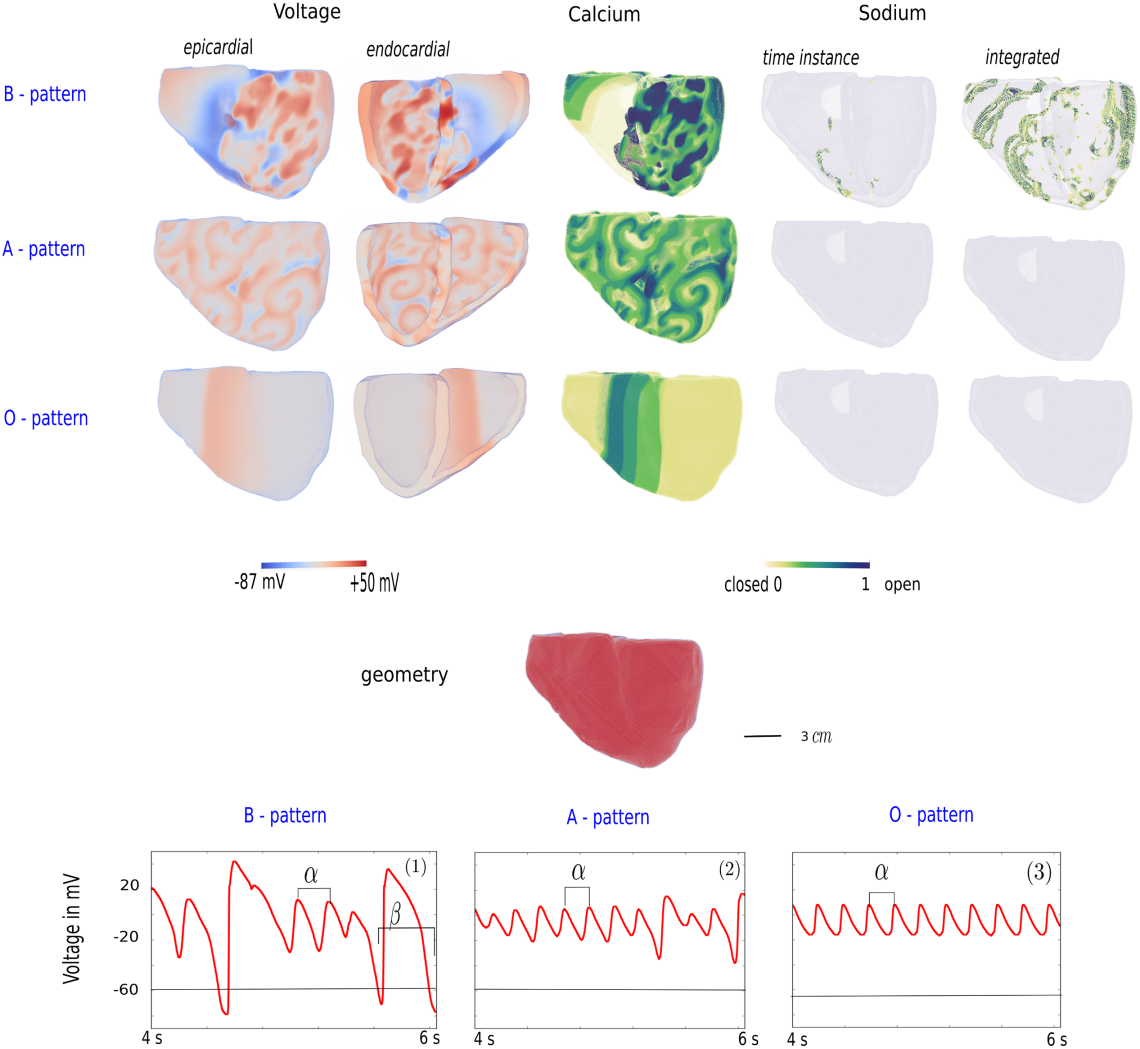
Electrophysiological behavior of different EAD regimes. In the first three rows, we illustrated the different electrophysiological features of the three patterns. Parameter values for this figures are set to 0.6 * *G*_*Kr*_ and 4.0 * *G*_*CaL*_ for the B-type patterns, 0.6 * *G*_*Kr*_ and 6.0 * *G*_*CaL*_ for the A-type patterns and 0.6 * *G*_*Kr*_ and 6.5 * *G*_*CaL*_ for the O-type patterns. The voltage is shown both for the epi- and endocardial view of the 3D ventricles. Next, the activity of the Calcium and Sodium channel gates, as well as the integrated Sodium channel activity are presented. The bottom panel illustrated the corresponding APs for each pattern during the last two seconds of the burst pacing simulation.

The three main excitation patterns which were considered in this paper are spiral fibrillation type B, spiral fibrillation type A and the oscillatory type O. In all cases, upon reduction of RR, we first obtained B, then A, and then O excitation patterns (see Fig 1). Starting from a simulation in a certain parameter range, we noticed that the development of the excitation patterns started in the intense paced regions, at the edge of the applied S1 pulse or in the core of the scroll wave. After further simulation, patterns spread across the 3D ventricles. Fig 2 illustrates the voltage patterns from the endo- and epicardial view and the opening of the Sodium and Calcium channel gates. To illustrate the opening of the gates of the Sodium current, this current was not only plotted for a time instance, but also the integrated current is shown. Also, for each pattern, a typical AP is drawn. The **B type** patterns were complex spatial-temporal patterns sustained by many short-lived spirals, which did complete a 360 degree rotation and chaotic waves which burst onto each other. In normal conditions, excitation waves in cardiac tissue are solely driven by Sodium mediated waves. However, from Fig 2 it was clear that the 3D ventricles was in a bi-excitable state where waves were not only mediated by Sodium, but also by Calcium [19]. During the AP, the voltage still reached -60 mV (black line), which is approximately the threshold for the Sodium channels to recover. However, the EADs did not reach this threshold and therefore created purely Calcium-mediated waves. An illustration of the B excitation patterns is shown in S1 Movie. The activation of the Sodium channels is illustrated in S2 Movie. Second, the **spiral fibrillation type A** was less chaotic than the B pattern, due to multiple small rotating spirals and waves which individually excited smaller parts of the 3D ventricles (S3 Movie). The spiral waves were the result of multiple wave breaks and even if the first break appeared in a complete different region of the 3D ventricles, the final state for all patterns in the category of A were similar. Depending on the position in the parameter domain of A, spirals were smaller in size (close to the parameter values of B) or larger (close to the parameter values of O). Therefore, more spirals were visible at the B side of the parameter range. Also compared to the B patterns, the 3D ventricles tissue in between the spirals repolarized less efficient, as could be seen by the less intensive blue colored regions in the A patterns versus the B patterns. Therefore, the waves for the A excitation patterns were solely mediated by Calcium-waves and thus the patterns are not formed due to the normal mechanisms of excitation in which the Sodium channel gates open, followed by normal APs. Hence, the Sodium and integrated Sodium-current (see Fig 2) showed no activity. Also, typical in the A patterns was the alternating AP formed by small and large EADs. Finally, the **O type** patterns have no repolarizing cells. Each AP oscillates around a higher state equilibrium resulting in a very small amplitude of the voltage. These oscillations were very regular, in contrast to the alternating APs of the B and A patterns. The O type patterns are constructed by repetitive passing of the S1 pulse as if it is applied multiple times. However, this is the result of the oscillating behavior of the cells as the threshold for a second pulse, S1 or S2, was never reached. A movie of the oscillatory pattern is shown in S4 Movie. Qualitatively, we found the same features as in 2D [21]. A quantitative analysis of the patterns was presented in the next sections.

**Fig 3:**
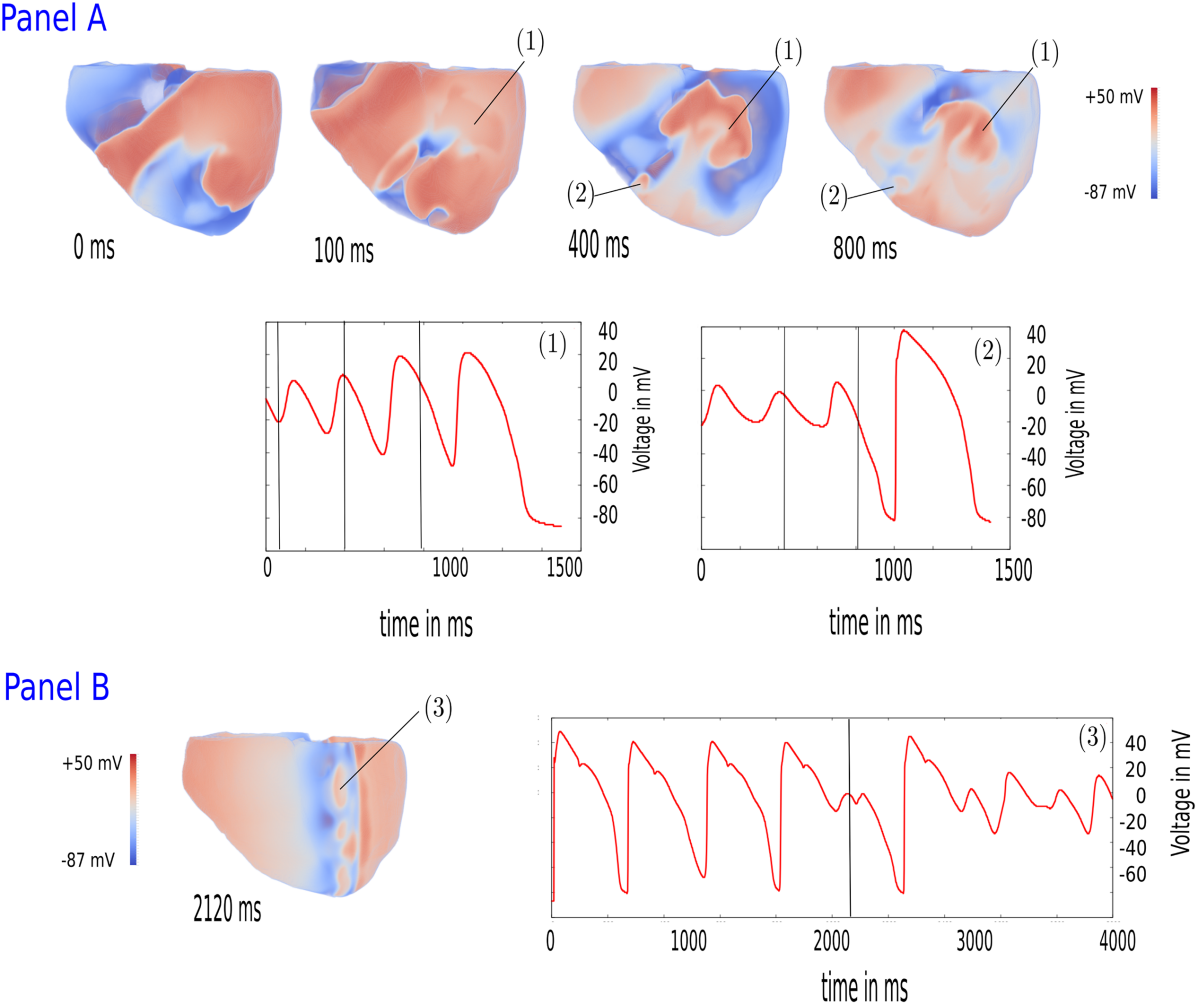
Formation of new sources due to EADs. Panel A: Dynamics of a spiral wave after changing the parameter values from 0.0 * *G*_*Kr*_, 2.0 * *G*_*CaL*_ to 0.0 * *G*_*Kr*_, 2.1 * *G*_*CaL*_. At the relative time instance of 100 ms, an EAD region is formed (denoted with (1)). A second EAD region emerged at the relative time instance of 400 ms (2). Both EAD regions disturbed the spiral wave dynamics and prohibited normal continuation of the wavefront. At 800 ms, the EAD regions created new sources of wave-formation and the B excitation pattern started to form gradually. Panel B: Formation of an EAD region at the back of the propagating wavefront during the burst pacing simulation at 2120 ms (3). The inserted graphs show electrical activity at points (1), (2) and (3).

In all cases, the formation of the patterns was due to EADs. Illustrated in Fig 3, we showed the formation of B patterns for both protocols (Panel 1 and Panel 2). For the S1S2 protocol (Panel 1, parameter values 2.1 * *G*_*CaL*_ and 0.0 * *G*_*Kr*_), the formation of additional EAD waves is shown at the locations marked by (1) and (2). First, indicated by (1), a local EAD cluster emerged in the tissue (t = 100 ms). As a result of the interaction of the original spiral wave with these synchronized EADs, we saw the formation of an additional spiral, moving in the opposite direction (t = 400 ms). At a different location in the 3D ventricles, marked by (2), another cluster of EADs emerged clearly at t = 400 ms. Due to the asynchronous behavior of these EADs on the spiral wave, the spiral started to break up in this region. Similarly, in the burst pacing protocol, see Fig 3 (parameter values 0.6 * *G*_*Kr*_ and 4.0 * *G*_*CaL*_), we saw formation of multiple EAD waves in the tail of the propagating wave (marked by (3)). APs in this tissue clearly showed EADs with large amplitudes. The time instance at t = 2120 ms was shown and denoted with the vertical line in the graph. Previous small EADs (e.g. at t = 1250 ms) did not form such EAD excitation waves. As in the case of the S1S2 protocol, interaction of the EAD waves with the initial waves formed new short lived spirals leading to the B pattern. Similarly, development of A and O patterns was always the result of the interaction of primary waves with EADs. Even though we homogeneously reduced the RR across the model, certain places were more likely to develop EADs and not all EADs emerging in the tissue resulted into wave breaks.

### Pattern characteristics

In the next sections, we characterized the observed patterns using several approaches.

#### Fourier transform

**Fig 4:**
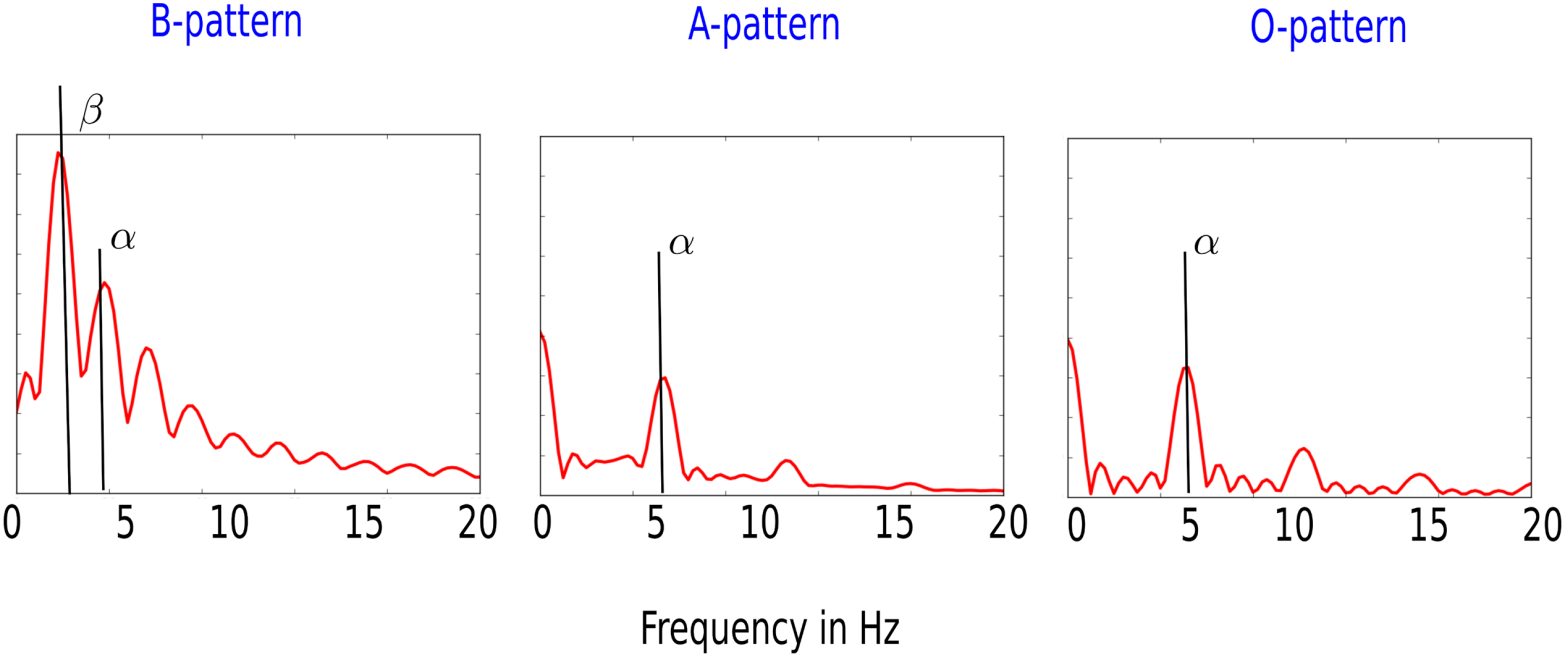
The average Fourier transform of transmembrane potential for the different activation patterns. The graph shows the average Fourier transform intensity for recordings at 2983 points during the last second of the simulations. Excitation pattern of type B was characterized by two main peaks: the larger peak *β* (agreeing with average distance between the APs) and smaller peak *α* (agreeing with average distance between the EADs). For excitation pattern of type A, the *α* peak became more pronounced than the *β* peak. For the excitation pattern O, the Fourier transform showed a clear *α*-peak at 5 Hz. Parameter values for above graphs are (0.6 * *G*_*Kr*_, 4.0 * *G*_*CaL*_) for the B, (0.6 * *G*_*Kr*_, 6.0 * *G*_*CaL*_) for the A and (0.6 * *G*_*Kr*_, 6.5 * *G*_*CaL*_) for the O excitation pattern.

The first analysis we applied was the average Fourier transform. To globally characterize the pattern, we computed the temporal Fourier transform of the AP of 2983 equidistant mesh nodes. The average Fourier transform for the burst pacing protocol is shown in Fig 4. The S1S2 protocol resulted in qualitatively similar graphs. For O excitation patterns (graph (3)), the *β*-peak was at 5 Hz corresponding to the period of 200 ms. For B excitation patterns, we saw prominent peaks at 2 Hz (*α*) and 4 Hz (*β*) corresponding to a temporal period of 250 and 500 ms. These periods agree with the average distance between two repolarized APs and two smaller oscillations of the EADs shown in Fig 1 (lower panel, graph (1)). These results resemble strongly the results of [21]. When the *α*-peak of the average Fourier transform was more pronounced then the *β*-peak was classified as pattern B. This is because the *α*-peak corresponds to the large amplitude AP characteristic of B patterns (Fig 1, lower panel, graph (1)).

#### Analysis of the AP

**Fig 5:**
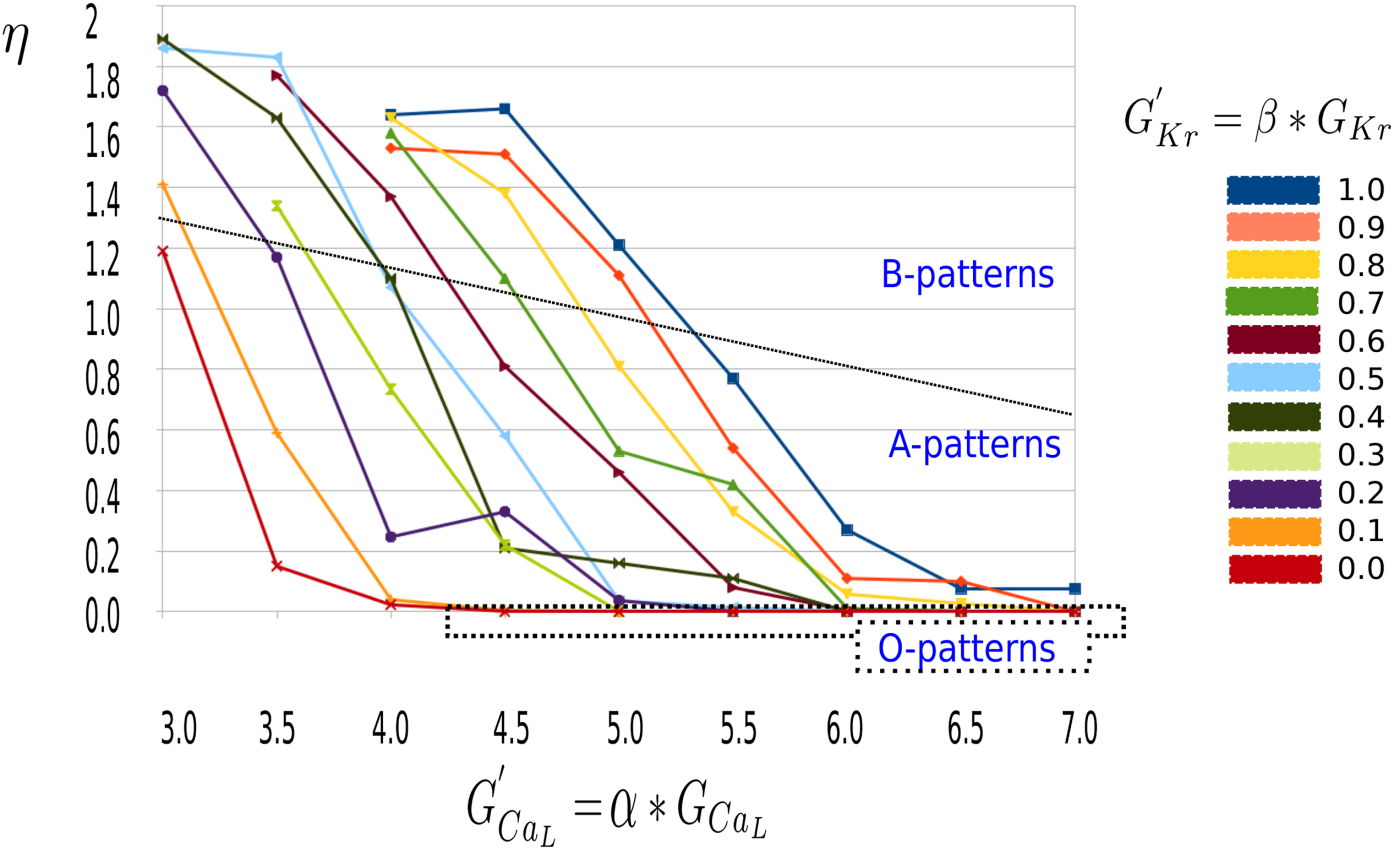
Statistical index *η* as a function of *I*_*CaL*_ for different values of *I*_*Kr*_. The calculation of *η* for a threshold of -60 mV and the last second of the burst pacing simulation. Different colors represent results for different values of *I*_*Kr*_. The decrease of *η* corresponded to the gradual shift of activity from Sodium to Calcium driven waves. A value *η* = 0 corresponds to the O-type excitation patterns, as is illustrated by the dotted box. The solid line separates the B patterns from the A patterns.

As shown in Fig 2, the main difference between A and B excitation patterns is the absence of the Sodium current. Sodium is inactivated as the diastolic potential is above the inactivation potential for Sodium channel gates which is approximately at −60 mV. Hence to quantify the type of waves in the patterns, we introduced a statistical index *η* given by:

$$\eta =\frac{x}{2n}$$ (5)

with x, how often the AP passed the voltage line of (-60 ± 1 mV) during the last second of the burst pacing simulations in the 2983 equidistant mesh nodes of the 3D ventricles. We divided the result by 2 to take into account the upstroke and down stroke of one AP. If at each point, the AP went below −60 mV, *η* corresponded with $\frac{time_{frame}}{period}$. For example for 1 cell during 1 sec simulation and 5 full normal APs, *η* was equal to 5. However for B patterns which completed repolarization, a lower value of *η* indicated that not all cells are stimulated by Sodium mediated waves and hence patterns also emerged due to Calcium mediated activation. A decrease of *η* hence indicated the transition between a bi-excitable state, in which both Calcium and Sodium waves mediated the pattern to a pure Calcium driven pattern. The overall data presented in Fig 5 showed indeed a gradually change from B to A with a decrease in the RR (by either increase of Calcium, or decrease of Potassium). Parameter values of O excitation patterns, presented in Fig 1, corresponded with a statistical average *η* of zero. For completeness, we present the standard deviation on the statistical average in S1 Table.

#### Phase mapping

**Fig 6:**
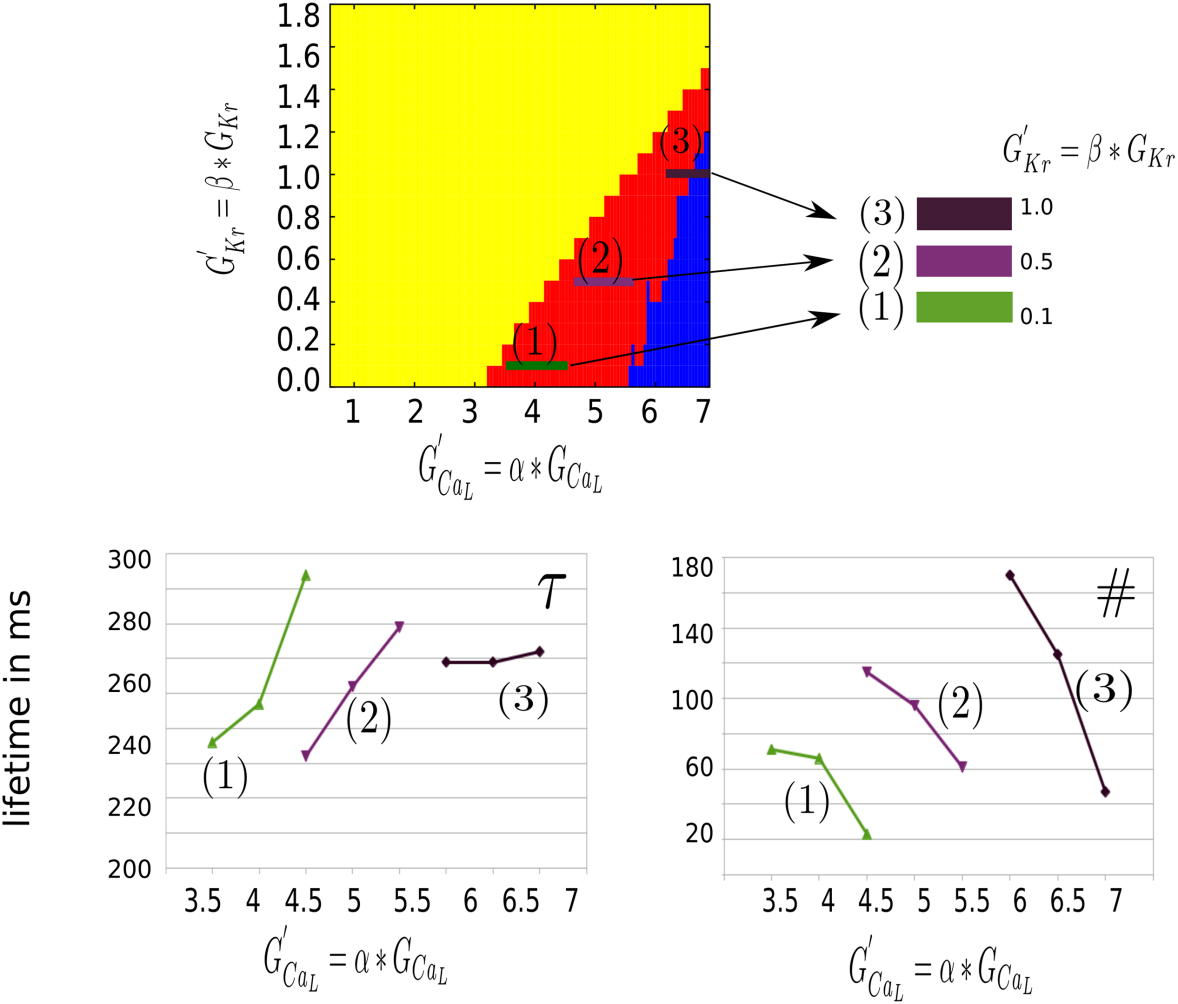
Number and lifetime of spirals in the A excitation patterns. The top panel illustrates the localization of the A excitation patterns in 2D parametric space. The colors correspond to the single cell simulations of [21]: yellow denotes AP with complete repolarization, red denotes AP with EADs but where repolarization still appears and blue denotes AP where there is no repolarization and the AP oscillates around a higher equilibrium state. (1), (2) and (3) correspond to A excitation patterns for *G*_*Kr*_ equal to 0.1, 0.5 and 1.0 times default, respectively. In the left bottom panel, the lifetime *τ* of the filaments is illustrated. For each value of *G*_*Kr*_ an increase of lifetime is visible with an increase of *G*_*CaL*_. The right bottom graph illustrates the corresponding decrease of the amount of spirals when *G*_*CaL*_ is increased at fixed *G*_*Kr*_.

Apart from the AP features, which were visible at the cell level, the spatial dynamics of the excitation patterns differed significantly. B type patterns were more chaotic and spiral waves did not finish a 360 degree rotation. A patterns however, showed multiple spirals with a clearly defined core. We counted the number of these (Calcium mediated) spirals with the help of phase-mapping and tracked the found singularities in time. We calculated the number (#) and lifetime (*τ*) of the filaments (A excitation patterns, burst pacing protocol, last second of the simulation). The results were shown in both Fig 6 and in S2 Table. In Fig 6, we noticed that for all values of *I*_*Kr*_ the lifetime of the spirals increased while the number of spirals decreased with an increase of *G*_*CaL*_. As increase in *G*_*CaL*_ decreases RR, we concluded that the reduction of RR increased the stability of the pattern and reduced its complexity.

#### Phase waves

**Fig 7:**
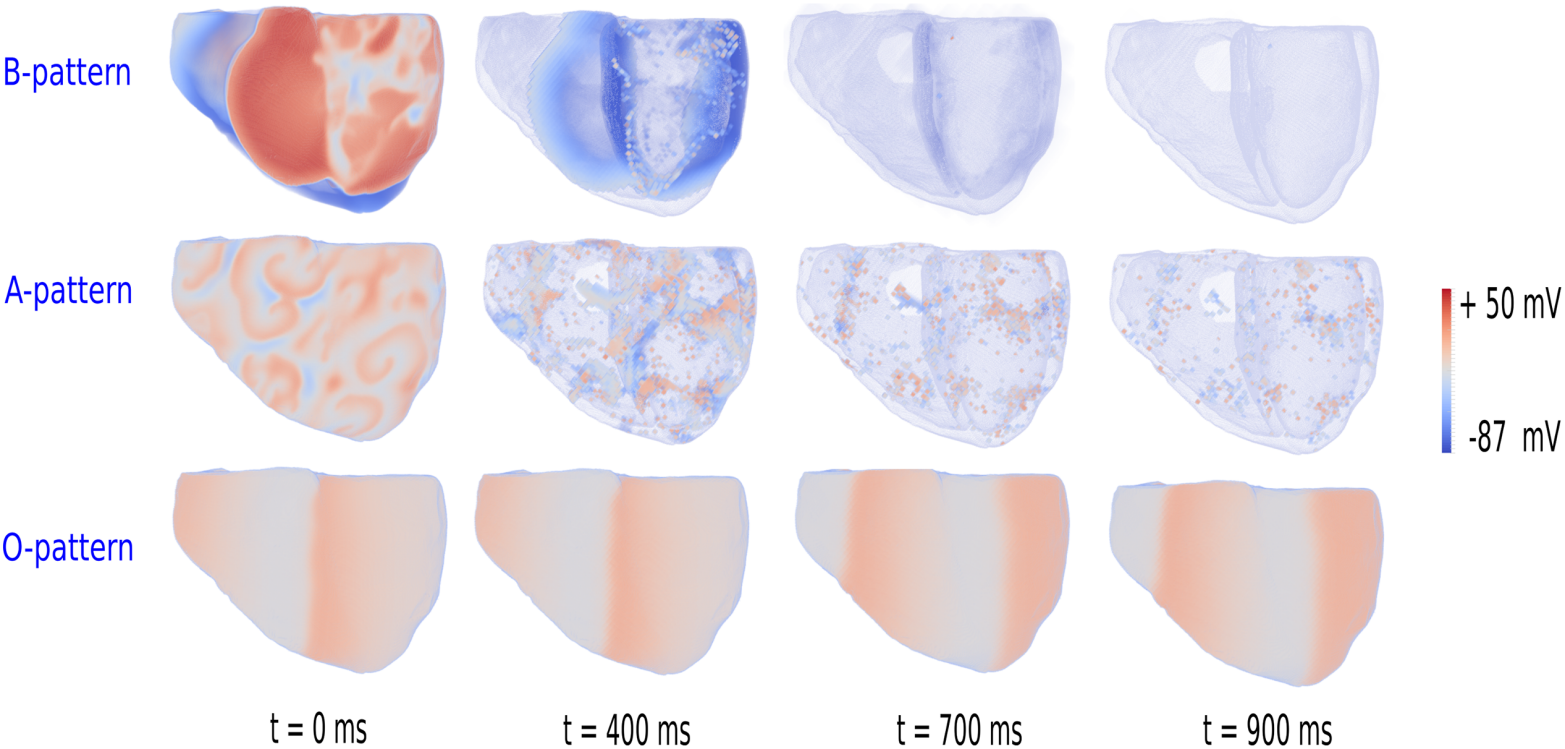
Phase waves versus real waves. Decoupling of the tissue exposed the real wave nature of B and A type pattern, while the O type patterns were clearly not effected by the impermeable walls, and are therefore consistent of phase waves. The indicated times denote simulation time after applying the impermeable walls.

As in [21], we disconnected the tissue along orthogonal planes to distinct between A and O patterns. These disconnections or impermeable walls prohibited diffusion between neighbouring cells hence exposing the nature of the waves: phase waves or real waves. Real waves are defined as conventional activation waves. They arise due to the interplay of the excitability of the tissue and diffusion. Real waves travel with a velocity proportional to the square root of the diffusion constant. At impermeable boundaries they are absorbed and do not go through regions in which the tissue is in a refractory state [49]. Phase waves however are determined by the pseudo-traveling character they possessed and often occur in oscillatory media [49]. They do not depend on the diffusion constant and are not hindered by impermeable boundaries. The results of the simulations are shown in Fig 7. We saw that for the B and A patterns, the excitation waves were hindered by the walls and eventually disappeared, and thus were mediated by normal waves. However, as expected, in the oscillatory regime, the walls did not affect wave propagation, and therefore patterns were mediated by phase waves. These simulations were run on the final state of the burst pacing and S1S2 protocol (after 6s and 5s simulation time, respectively).

### Including a torso model: Interpretation of the patterns using ECGs

**Fig 8:**
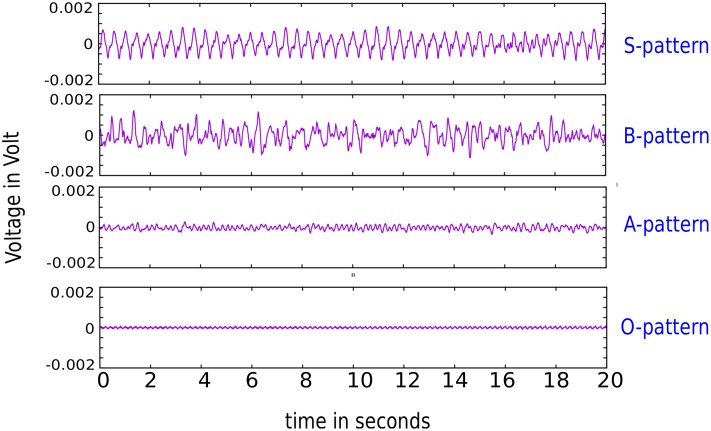
Lead II of the ECG for the patterns of the stable spiral (1.0 * *G*_*Kr*_, 0.5 * *G*_*CaL*_), B (0.6 * *G*_*Kr*_, 4.0 * *G*_*CaL*_), A (0.6 * *G*_*Kr*_, 5.5 * *G*_*CaL*_) and O (0.6 * *G*_*Kr*_, 6.5 * *G*_*CaL*_) excitation patterns. The stable spiral produced a typical VT ECG. B excitation patterns produced ECGs resembling VT whilst A excitation patterns were clearly VF. The oscillatory pattern showed a regular ECG with small amplitude, due to the regularity of the APs.

After classification of the patterns, we calculated the realistic 9-lead ECGs. We simulated the patterns for 20 seconds starting from the final state (after 6 or 5 seconds for burst pacing or S1S2, respectively). With a gradual increase of *G*_*CaL*_, the amplitude of the ECG signal decreased in accordance with the decreasing scale of APs. For comparison, we plotted the ECG II lead signal for the stable spiral together with the ones for the B, A and O excitation type in Fig 8. These patterns corresponded to parameter values of 1.0 * *G*_*Kr*_ and 0.5 * *G*_*CaL*_, 0.6 * *G*_*Kr*_ and 4.0 * *G*_*CaL*_, 0.6 * *G*_*Kr*_ and 5.5 * *G*_*CaL*_ and 0.6 * *G*_*Kr*_ and 6.5 * *G*_*CaL*_, respectively. First, we saw that a stable spiral gave rise to a VT, as is well known. Also the B type resembled a PVT while the A type was clearly related to VF. The oscillatory pattern gave a regular ECG, due to the regular nature of all its APs. All 9 ECG leads for the B, A and O patterns were shown in S4, S5 and S6 Figs.

## Discussion

This study has focused on different spatial patterns due to EADs on an anatomically accurate model of the human ventricles. It considered homogeneous ventricles with single cell equations of the Ten Tusscher Panfilov model [23, 24]. EADs were induced by reduction of the repolarization reserve by either lowering the rapid delayed rectifier Potassium current *I*_*Kr*_ and/or raising the L-type Calcium current *I*_*CaL*_. This created a 2D parametric space in which the different patterns were located. We generated the patterns with two different protocols: burst pacing and S1S2 pacing. As in 2D [21], we found three different types of patterns: spiral fibrillation type B, spiral fibrillation type A and an oscillatory pattern. The B pattern showed bi-stable waves, similarly to [19], indicating that the waves were Calcium- and Sodium mediated and co-existed in the same medium. This pattern was chaotic, comprising multiple short-lived spiral waves, illustrated in S1 Movie. The waves were actively propagating, as impermeable walls absorbed the waves. The average temporal Fourier transform of multiple mesh nodes of the ventricles showed waves of large periods around 500 ms, corresponding to the period of one complete de- and repolarization of one cell. Also a characteristic frequency of 4 Hz was found, corresponding to the 250 ms period generated by the EADs. This period deviates strongly from the period found in [19]. In this study, the bi-excitability of the tissue showed both Sodium and Calcium waves maintaining the spiral. In the Calcium mediated core of that spiral, the period corresponded to around 500 ms.

The A type was formed due to the interaction of multiple rotating spirals. In contrast to the patterns of B, the A waves were mostly mediated by Calcium waves. Similarly, they were absorbed by the impermeable walls. The probability of complete re-polarization after multiple small EADs in the AP of the cells decreased upon reduction of RR. Also, the behavior of the spirals changed when repolarization reserve was decreased: it resulted in less number of spirals and their longer life-time. Finally, the oscillatory pattern in contrast to waves mediating the B and A type, were phase waves. Impermeable walls did not hinder the observed wave dynamics. We have also performed the calculation of realistic 9-lead ECGs. Here, we found that none of the 3 described patterns showed the typical Torsade de Pointes (TdP) characteristics with the common twisting of the ECG around the iso-electrical baseline [50]. The common TdP like varying amplitude was only seen in the meandering spiral MS just before the spiral started to break up. This interesting phenomenon will however be the topic of a subsequent study. For current patterns, ECG signals showed the typical form of (p)VT for the B excitation patterns and a VF signal for the A excitation patterns. Accordingly with the decreased amplitude of the AP, the amplitude of the ECG signal of A excitation patterns was remarkably reduced. The oscillatory pattern is not likely to occur spontaneously due to homogeneous and highly abnormal values of the adapted gates and according ECG signals did not correspond to realistic recordings. However, this latter pattern can be interesting, as it can be possible that a small heterogeneity in the 3D ventricles is in the parameter range of this pattern. In that case, this heterogeneity will emit ectopic beats and in presence of multiple heterogeneities of this type, they can have complex interactions with each other and form the mechanism of TdP, perpetuated by ectopic beats [30].

Even though it is well established that EADs are associated with an increased arrhythmogeneity, only one study previously investigated the effects of EADs in a homogeneous 3D ventricles [16]. However, the underlying single cell dynamics was represented by a rabbit ventricular model. In [16] it was found that EADs can locally cluster to form islands of EADs which were locally synchronized and emitted new waves. The pattern found in that study showed similarities with the B pattern of our study and the ECG created by these patterns resembled the ECG of PVT like our B excitation pattern. Different is however that those patterns stopped spontaneously, what we did not observe in the current study.

### Limitations

During this research we have only used the TP06 model of the human ventricular cardiac cell in a 3D ventricles with a certain anisotropy. Also the 3D ventricles did not include electrophysiological heterogeneities. It would therefore be interesting to study this further with different cell models.

Secondly we remark that we only studied the patterns for homogeneous tissue. However, realistic cardiac tissue is heterogeneous, with different types of cells like epi, endo and midmyocardial cells. The effect of heterogeneities was partially studied in [30], where the role of small sized heterogeneities similar to those found experimentally by Glukhov et al. [32] was investigated. In [30] it was found such heterogeneities can initiate ectopic activity and fibrillatory patterns of excitation depending on the repolarization reserve at the heterogeneities and at the surrounding tissue. However there were no detailed studies of effects of gradient heterogeneities on the organization of the excitation patterns in EAD prone tissue. it would be interesting to perform such studies and investigate if presence of such heterogeneities will result in additional complexity of the excitation patterns. It would also be interesting to study how presence of EADs affects defibrillation of the arrhythmias [51, 52]. Also, as arrhythmias occur at different heart rates, we could also investigate the pacing dependency of the patterns on the applied S1 pulse. However in 2D [21], this was not the case and the final patterns were qualitatively the same. Finally, we did not study the effect of the Purkinje-His system on the pattern formation. However, as is well known, the Purkinje cells exhibit EADs more easily [53, 54] and could therefore add another layer of complexity to the development of the established patterns. This question is however beyond the scope of the current paper as we wanted to study types of patterns in the most basicsetup of homogeneous systems. We also study developed patterns of arrhythmias which occur after all transient processes. Because the Purkinje cells have longer refractory period [53, 54] it is less likely that they will affect established excitation patterns, however they may be important at the initial stage of arrhythmia onset.

### Future study

Using the S1S2 protocol, we found in the parameter regions before the B type patterns started meandering spirals and upon further raising the L-type calcium current, spirals which broke up slightly into two competing spirals. EADs were responsible for the meandering and the break-up. We will therefore investigate these interesting patterns and their behavior on the ECG which might resemble the signals of TdP in a subsequent study.

## Supporting information

S1 TableValues including the standard deviation of the statistical index *η* as a function of $G_{CaL}^{‘}$ for different values of $G_{Kr}^{‘}$, see also Fig 4.We increased L-type calcium in the different columns: $G_{CaL}^{‘}=\alpha *G_{CaL}$. Different rows denote a different $G_{Kr}^{‘}=\beta *G_{Kr}$.(PDF)

S2 TableFilaments lifetime and corresponding standard deviation in ms for the A excitation patterns, see also Fig 5.We increased L-type calcium in the different columns: $G_{CaL}^{‘}=\alpha *G_{CaL}$. Different rows denote a different $G_{Kr}^{‘}=\beta *G_{Kr}$.(PDF)

S1 Fig Simulated Sinus Rhythm (SR) in the 3D model of the ventricles. In this figure, we see that total excitation required 96 ms in accordance with detected duration of the QRS complex in patients.
(PDF)

S2 Fig Comparison of excitation patterns for a spatial resolution *dx* = 0.4 mm and *dx* = 0.2 mm for parameter values representative for the B, A and O excitation pattern in the heart wedge preparation. First, the excitation patterns look similar for both integration steps. Second, the temporal Fourier transform for the B and A patterns show the same characteristic *α* and *β* peaks, which were used to differentiate between the B and A excitation patterns of the ventricles. The decoupling of the tissue along orthogonal planes, results in a continuation of excitation for the O pattern and disappearance of excitation for the A (and B) excitation patterns.
(PDF)

S3 Fig Pattern evolution with an increase of *G*_*CaL*_ at fixed *G*_*Kr*_. The complexity of the spatio-temporal pattern increased due to the increase of *G*_*CaL*_ from 2.0-fold to 7.0-fold. The meandering spiral at 2.0-fold broke up and eventually resulted into an B pattern for *G*_*CaL*_ = 2.5, 3.0 times default. Further increase resulted in a continuously changing pattern from B into A (for *G*_*CaL*_ = 3.5, 4.0, 4.5 times default) to O (for *G*_*CaL*_ = 5.0–7.0 times default). The number in the figure denoted the increase *G*_*CaL*_-value, while *G*_*Kr*_ = 0.
(PDF)

S4 FigECG of B excitation pattern.9 out of the standard 12 ECG leads for the B excitation patterns. Result of a 10 second simulation after the 6 seconds simulation time in which patterns were created and evolved. The parameter values were set to $G_{Kr}^{‘}=0.6*G_{Kr}$ and $G_{CaL}^{‘}=4.0*G_{CaL}$. The ECG shows the signature of VT.(PDF)

S5 FigECG of A excitation pattern.9 out of the standard 12 ECG leads for the A excitation patterns. Result of a 10 second simulation after the 6 seconds simulation time in which patterns were created and evolved. The parameter values were set to $G_{Kr}^{‘}=0.6*G_{Kr}$ and $G_{CaL}^{‘}=5.5*G_{CaL}$. The ECG shows the signature of VF.(PDF)

S6 FigECG of O excitation pattern.9 out of the standard 12 ECG leads for the O excitation patterns. Result of a 10 second simulation after the 6 seconds simulation time in which patterns were created and evolved. The parameter values were set to $G_{Kr}^{‘}=0.6*G_{Kr}$ and $G_{CaL}^{‘}=6.5*G_{CaL}$. The regularity of the ECG is explained by the regular oscillatory nature of the single cells.(PDF)

S1 Movie Movie of the B excitation pattern. Voltage pattern of the B excitation pattern.
(OGV)

S2 Movie Movie of the B-sodium gates. Illustration of the activation of the Sodium gates of the B excitation pattern.
(OGV)

S3 Movie Movie of the A excitation pattern. Voltage pattern of the A excitation pattern.
(OGV)

S4 Movie Movie of the O excitation pattern. Voltage pattern of the O excitation pattern.
(OGV)

## Abbreviations

A: Spiral Fibrillation type A
B: Spiral Fibrillation type B
EAD: Early Afterdepolarization
ECG: Electrocardiogram
EE: Excitation Ended
MS: Meandering Spiral
O: Oscillatory Fibrillation type
ORD: Ohara Rudy mathematical model
PF: Purkinje Fiber
RR: Repolarization Reserve
s1pacing: S1 pulse burst pacing protocol
s1s2: Spiral Inducing protocol
SB: Spiral Breakup
SR: Sinus Rhythm
SS: Stable Spiral
TdP: Torsade de Pointes
TP06: Ten Tusscher—Panfilov mathematical model for human ventricular cells (2006)
VF: Ventricular Fibrillation
VT: Ventricular Tachycardia
