# Supplementary material for: Effects of early afterdepolarizations on excitation patterns in an accurate model of the human ventricles
Source: PLoS One. 2017 Dec 7;12(12):e0188867. doi: 10.1371/journal.pone.0188867 (PMC5720514; doi:10.1371/journal.pone.0188867)
Supplement: S1 Table — We increased L-type calcium in the different columns: GCaL‘=α*GCaL. Different rows denote a different GKr‘=β*GKr. (PDF) [file pone.0188867.s001.pdf]

Table 1. Values including the standard deviation of the statistical index η as a function of $G^{'}_{CaL}$ for different values of $G^{'}_{Kr}$, see also Fig. 4. We increased L-type calcium in the different columns: $G^{'}_{CaL} = \alpha * G_{CaL}$. Different rows denote a different $G^{'}_{Kr} = \beta * G_{Kr}$.

	3.0	3.5	4.0	4.5	5.0	5.5	6.0	6.5	7.0
1.0			1.64 ± 1.38	1.66 ± 1.49	1.21 ± 1.75	0.77 ± 1.57	0.27 ± 1.0	0.076± 0.55	0.076 ± 0.51
0.9			1.53 ± 1.27	1.51±1.84	1.11±1.87	0.54±1.37	0.11±0.66	0.1±0.58	0±0
0.8			1.63 ± 1.33	1.38±1.76	0.81±1.58	0.33±1.13	0.058±0.43	0.027±0.32	0±0
0.7			1.58 ± 1.51	1.1±1.80	0.53±1.41	0.42±1.18	0.011±0.22	0±0	0±0
0.6		1.77 ±1.61	1.37 ±1.64	0.81±1.53	0.46±1.18	0.08±0.54	0.0045± 0.152	0±0	0±0
0.5	1.86 ± 1.69	1.83 ± 1.97	1.07±1.59	0.58±1.37	0.036±0.41	0.014±0.23	0±0	0±0	0±0
0.4	1.89 ±1.71	1.63±1.70	1.1±1.70	0.21±0.92	0.16±0.70	0.11±0.55	0±0	0±0	0± 0
0.3		1.34 ±1.59	0.734±1.43	0.22±0.73	0±0	0± 0	0± 0	0±0	0± 0
0.2	1.72 ±1.62	1.17±1.69	0.247±0.915	0.33±1.15	0.037±0.32	0±0	0± 0	0±0	0±0
0.1	1.41 ±1.53	0.59± 1.38	0.04±0.39	0.004± 0.1	0±0	0±0	0± 0	0±0	0± 0
0.0	1.19 ±1.54	0.15±0.7	0.023±0.26	0±0	0±0	0±0	0±0	0±0	0±0
