# Supplementary material for: Effects of early afterdepolarizations on excitation patterns in an accurate model of the human ventricles
Source: PLoS One. 2017 Dec 7;12(12):e0188867. doi: 10.1371/journal.pone.0188867 (PMC5720514; doi:10.1371/journal.pone.0188867)
Supplement: S2 Table — We increased L-type calcium in the different columns: GCaL‘=α*GCaL. Different rows denote a different GKr‘=β*GKr. (PDF) [file pone.0188867.s002.pdf]

Table 1. Filaments lifetime and corresponding standard deviation in ms for the A excitation patterns, see also Fig. 5. We increased L-type calcium in the different columns: $G^{`}_{CaL} = \alpha * G_{CaL}$. Different rows denote a different $G^{`}_{Kr}$ $= \beta * G_{Kr}$.

	3.5	4.0	4.5	5.0	5.5	6.0	6.5	7.0
1.0						512±136	512 ± 124	518 ± 124
0.9						506±132	530 ± 124	
0.8					494 ± 142	550± 100	550± 92	
0.7				438± 144	518± 122	534± 114		
0.6			464± 142	444± 156	526± 122			
0.5			458± 146	498± 144	532± 118			
0.4			486± 138	498±144				
0.3			520± 128	586±50				
0.2		462± 152	576 ±78	546±118				
0.1	466±150	488±136	562±96					
0.0	444±150	466± 166						
