# Supplementary material for: Effects of early afterdepolarizations on excitation patterns in an accurate model of the human ventricles
Source: PLoS One. 2017 Dec 7;12(12):e0188867. doi: 10.1371/journal.pone.0188867 (PMC5720514; doi:10.1371/journal.pone.0188867)
Supplement: S1 Fig — In this figure, we see that total excitation required 96 ms in accordance with detected duration of the QRS complex in patients. (PDF) [file pone.0188867.s003.pdf]

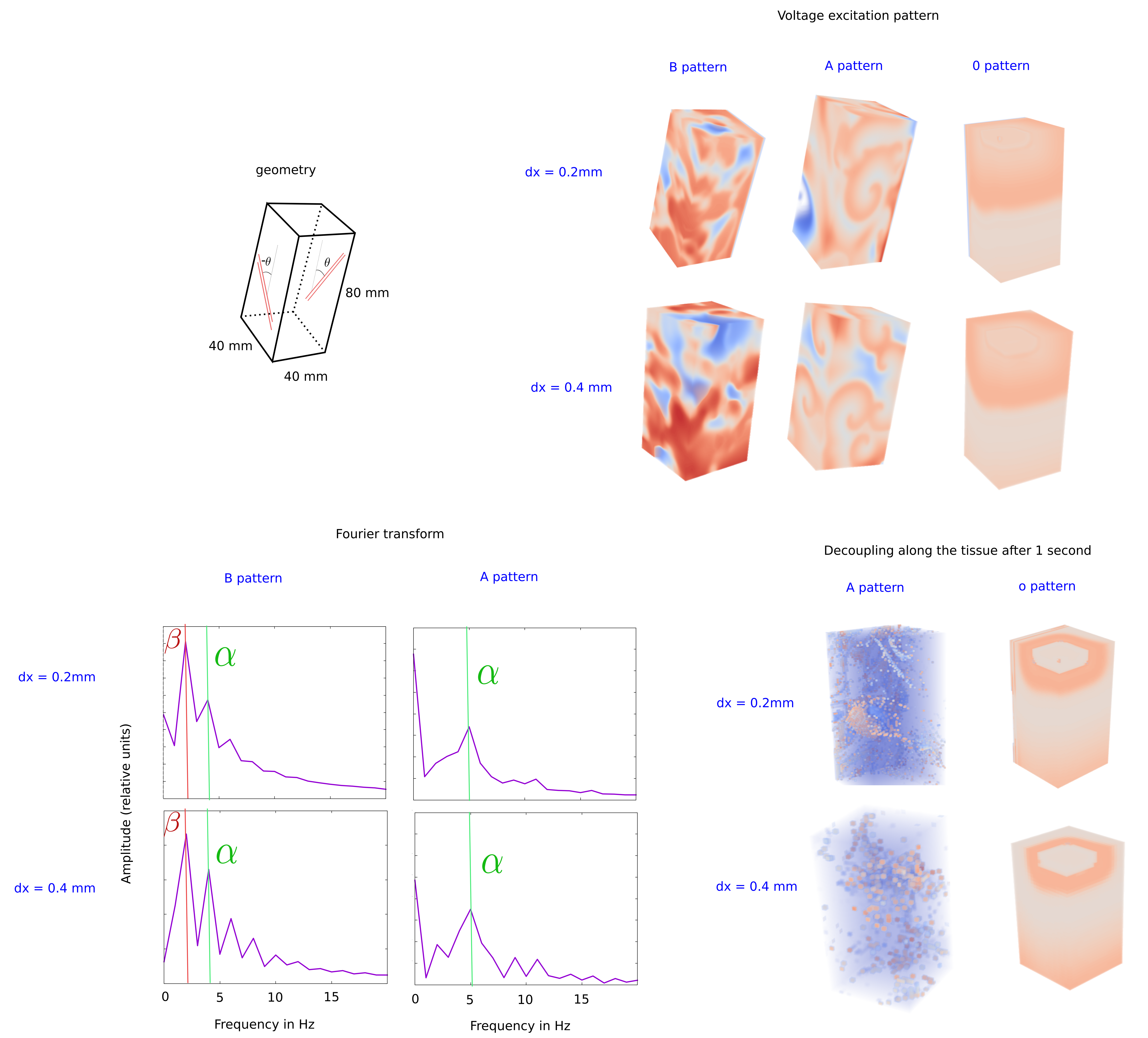

Voltage excitation pattern
B pattern
A pattern
0 pattern
dx = 0.2mm
dx = 0.4 mm
geometry
-θ
θ
80 mm
40 mm
40 mm
Fourier transform
B pattern
A pattern
dx = 0.2mm
dx = 0.4 mm
Amplitude (relative units)
β
α
0
5
10
15
Frequency in Hz
Frequency in Hz
Decoupling along the tissue after 1 second
A pattern
o pattern
dx = 0.2mm
dx = 0.4 mm
