# Supplementary figures and images for: Effects of early afterdepolarizations on excitation patterns in an accurate model of the human ventricles
Source: PLoS One. 2017 Dec 7;12(12):e0188867. doi: 10.1371/journal.pone.0188867 (PMC5720514; doi:10.1371/journal.pone.0188867)

50 mV  
-87 mV

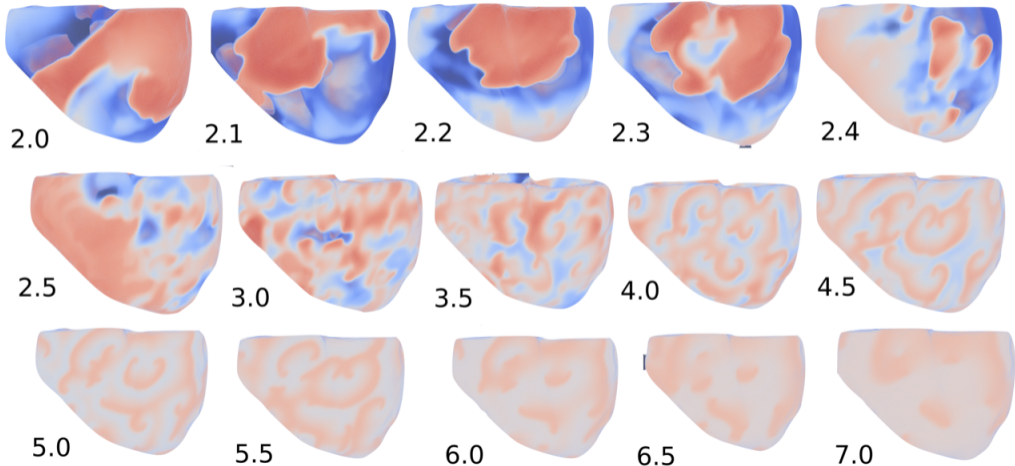

Supplement: S3 Fig — The complexity of the spatio-temporal pattern increased due to the increase of GCaL from 2.0-fold to 7.0-fold. The meandering spiral at 2.0-fold broke up and eventually resulted into an B pattern for GCaL = 2.5, 3.0 times default. Further increase resulted in a continuously changing pattern from B into A (for GCaL = 3.5, 4.0, 4.5 times default) to O (for GCaL = 5.0–7.0 times default). The number in the figure denoted the increase GCaL-value, while GKr = 0. (PDF) [file pone.0188867.s005.pdf]

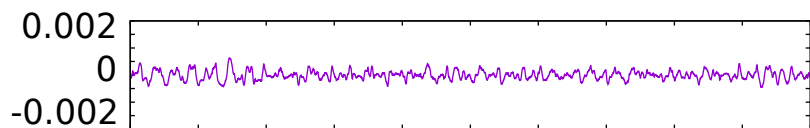

I

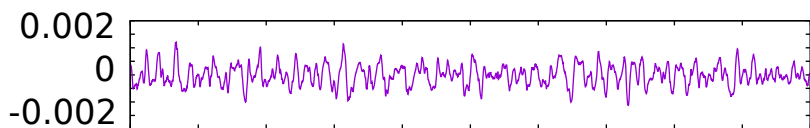

II

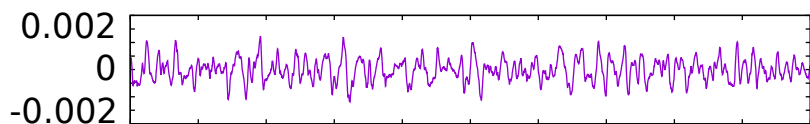

III

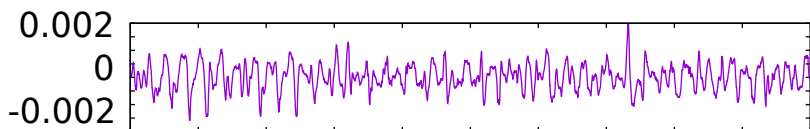

V1

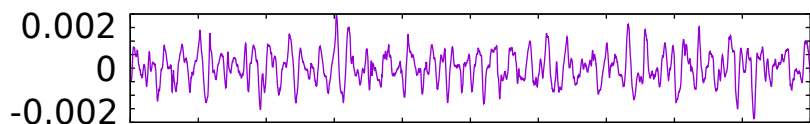

V2

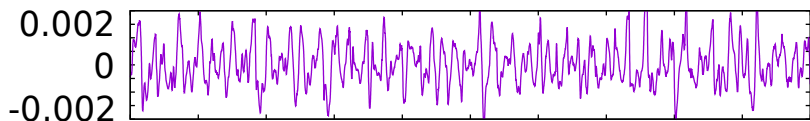

V3

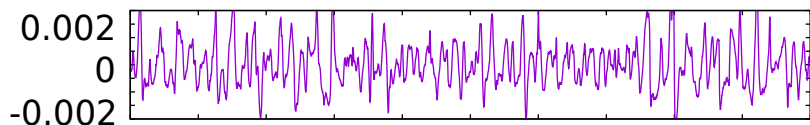

V4

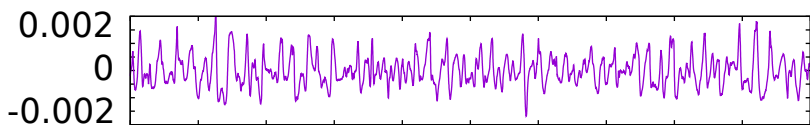

V5

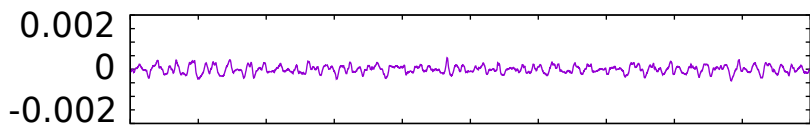

V6

0 2 4 6 8 10 12 14 16 18 20

time in seconds

Supplement: S4 Fig — 9 out of the standard 12 ECG leads for the B excitation patterns. Result of a 10 second simulation after the 6 seconds simulation time in which patterns were created and evolved. The parameter values were set to GKr‘=0.6*GKr and GCaL‘=4.0*GCaL. The ECG shows the signature of VT. (PDF) [file pone.0188867.s006.pdf]

Voltage in Volt

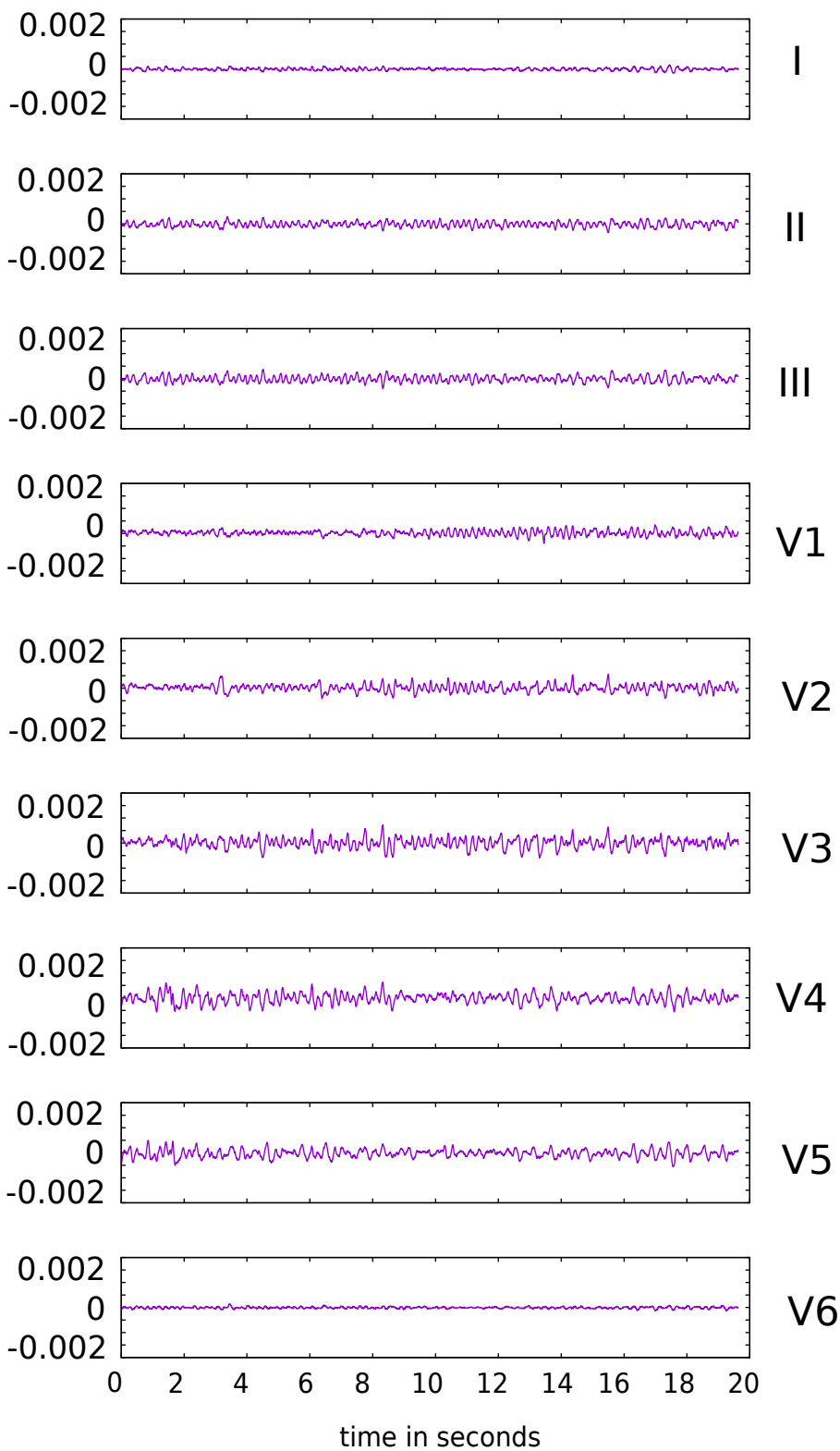

Supplement: S5 Fig — 9 out of the standard 12 ECG leads for the A excitation patterns. Result of a 10 second simulation after the 6 seconds simulation time in which patterns were created and evolved. The parameter values were set to GKr‘=0.6*GKr and GCaL‘=5.5*GCaL. The ECG shows the signature of VF. (PDF) [file pone.0188867.s007.pdf]

Voltage in Volt

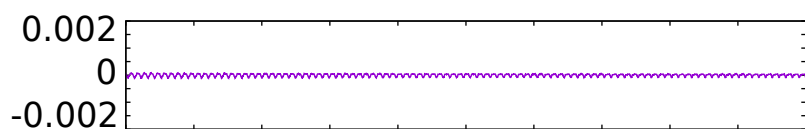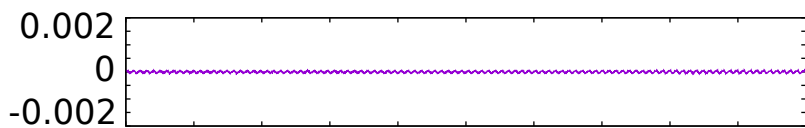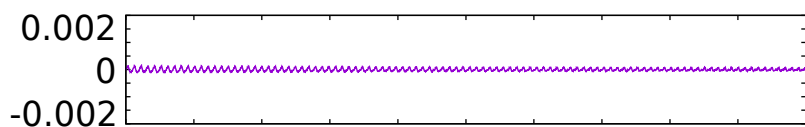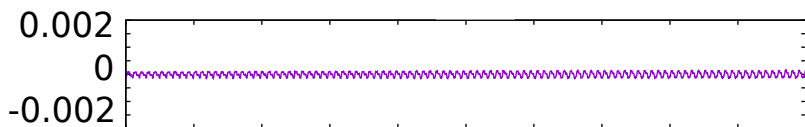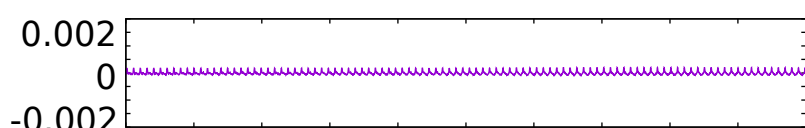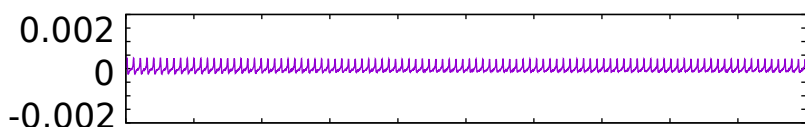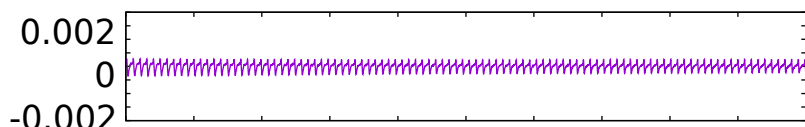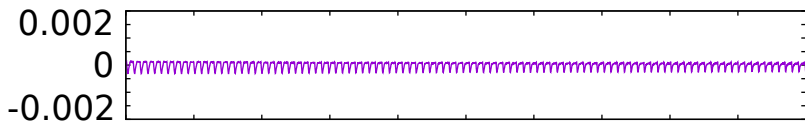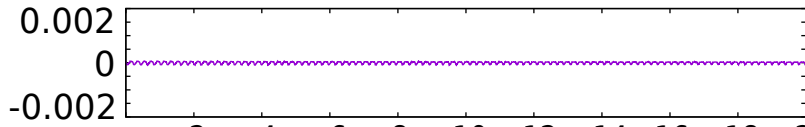

0 2 4 6 8 10 12 14 16 18 20

time in seconds

Supplement: S6 Fig — 9 out of the standard 12 ECG leads for the O excitation patterns. Result of a 10 second simulation after the 6 seconds simulation time in which patterns were created and evolved. The parameter values were set to GKr‘=0.6*GKr and GCaL‘=6.5*GCaL. The regularity of the ECG is explained by the regular oscillatory nature of the single cells. (PDF) [file pone.0188867.s008.pdf]
