# Supplementary material for: Effects of early afterdepolarizations on excitation patterns in an accurate model of the human ventricles
Source: PLoS One. 2017 Dec 7;12(12):e0188867. doi: 10.1371/journal.pone.0188867 (PMC5720514; doi:10.1371/journal.pone.0188867)
Supplement: S2 Fig — First, the excitation patterns look similar for both integration steps. Second, the temporal Fourier transform for the B and A patterns show the same characteristic α and β peaks, which were used to differentiate between the B and A excitation patterns of the ventricles. The decoupling of the tissue along orthogonal planes, results in a continuation of excitation for the O pattern and disappearance of excitation for the A (and B) excitation patterns. (PDF) [file pone.0188867.s004.pdf]

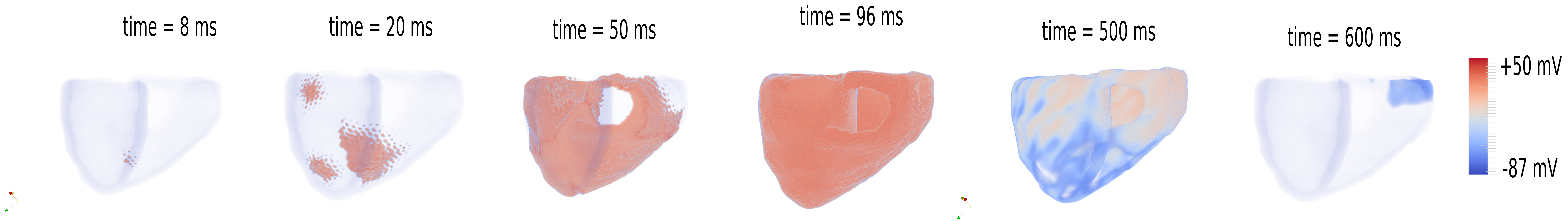

time = 8 ms
time = 20 ms
time = 50 ms
time = 96 ms
time = 500 ms
time = 600 ms
+50 mV
-87 mV
